# Controlled Soil Warming Powered by Alternative Energy for Remote Field Sites

**DOI:** 10.1371/journal.pone.0082903

**Published:** 2013-12-26

**Authors:** Jill F. Johnstone, Jonathan Henkelman, Kirsten Allen, Warren Helgason, Angela Bedard-Haughn

**Affiliations:** 1 Department of Biology, University of Saskatchewan, Saskatoon, Saskatchewan, Canada; 2 Department of Soil Science, University of Saskatchewan, Saskatoon, Saskatchewan, Canada; 3 Department of Chemical and Biological Engineering, University of Saskatchewan, Saskatoon, Saskatchewan, Canada; Tennessee State University, United States of America

## Abstract

Experiments using controlled manipulation of climate variables in the field are critical for developing and testing mechanistic models of ecosystem responses to climate change. Despite rapid changes in climate observed in many high latitude and high altitude environments, controlled manipulations in these remote regions have largely been limited to passive experimental methods with variable effects on environmental factors. In this study, we tested a method of controlled soil warming suitable for remote field locations that can be powered using alternative energy sources. The design was tested in high latitude, alpine tundra of southern Yukon Territory, Canada, in 2010 and 2011. Electrical warming probes were inserted vertically in the near-surface soil and powered with photovoltaics attached to a monitoring and control system. The warming manipulation achieved a stable target warming of 1.3 to 2°C in 1 m^2^ plots while minimizing disturbance to soil and vegetation. Active control of power output in the warming plots allowed the treatment to closely match spatial and temporal variations in soil temperature while optimizing system performance during periods of low power supply. Active soil heating with vertical electric probes powered by alternative energy is a viable option for remote sites and presents a low-disturbance option for soil warming experiments. This active heating design provides a valuable tool for examining the impacts of soil warming on ecosystem processes.

## Introduction

The global climate is changing and ecological systems are increasingly being exposed to climate conditions that are outside the historic norm [1]. Understanding and forecasting ecosystem responses to these changes is a key element required to inform societal responses to change in Earth's systems. However, we often lack direct experimental data on how altered climate conditions, such as warmer temperatures, influence ecosystem processes and community dynamics in real-world settings. Field experiments are critical to developing a predictive understanding of ecosystem responses because they permit explicit testing of causal processes. In situations where we expect ecosystems to experience novel environmental conditions, process-based experiments become essential for anticipating changes to ecosystem structure and function [2], [3].

Climate change is expected to happen most rapidly in high latitude regions [1] and substantial warming has already been detected in many arctic and alpine tundra environments [4], [5]. Previous research in tundra ecosystems indicate that vegetation composition and productivity are more sensitive to changes in soil nutrients than to warming of the air [6], [7], [8], [9]. However, microbial processes that control nutrient cycling are themselves strongly influenced by temperature [6], [8], [10]. Thus, tundra plant communities may be more responsive to indirect effects of warming on soil processes as compared to direct effects on photosynthesis and aboveground growth [8].

Despite the importance of soil processes in regulating plant communities, there are relatively few field experiments that apply realistic manipulations of soil climate [11], [12], [13], [14], and even fewer in high latitude or high altitude environments [15]. There are many technical challenges to implementing climate change experiments in the field, including reduction of unwanted environmental effects, implementation of sustained warming, and mimicking natural patterns of variability [15], [16], [17]. These challenges are particularly acute in remote regions at high latitudes or altitudes, where infrastructure support for field experiments is very limited. Consequently, climate change experiments in these areas often rely on simple, passive techniques, such as closed or open-topped greenhouses, to manipulate temperature conditions in experimental plots [15], [17], [18], [19]. However, passive warming designs may fail to achieve significant or consistent soil warming [20], [21], [22], [23], [24], [25], making them unreliable tools for investigating temperature effects on soil processes. Similarly, non-experimental approaches, such as studies across natural climate gradients (e.g. [26]), necessarily confound multiple biotic and abiotic factors and make it challenging in the absence of experimentation to assess sensitivity to specific climate factors [16].

Alternate approaches of active experimental warming, such as infrared heaters [13], [27] or buried soil heating cables [28], [29], [30], have been used to achieve consistent, controlled warming of surface soils in a range of ecosystem types. However, these methods of active warming require access to large amounts of electricity and are thus are not feasible in remote areas isolated from power sources. An additional complication is that burying heating cables disturbs soils and vegetation during installation [28], which is a particular concern in slow-growing tundra ecosystems. Consequently, there remains a need for efficient, minimally disruptive methods of controlled warming that can be applied in remote areas. Here we test the feasibility of achieving controlled soil warming in alpine tundra using solar-powered heating cables to warm near-surface soils with little disturbance to soil or vegetation. This approach has the potential to provide a robust, flexible, and relatively precise system for implementing soil warming experiments in natural settings, including remote regions without access to grid power.

## Materials and Methods

### Study Site

The experimental site was located in the alpine zone of the Wolf Creek drainage basin (N 60°33′46.4″, W 135°07′55.0″, elevation 1565 m), approximately 20 km south of Whitehorse, Yukon Territory. Permission for the research was obtained through the Yukon Territorial Government via Science and Exploration permit numbers 10-04 and 11–26. The area is characterized by mountainous terrain with boreal forest at low elevations and alpine tundra at higher elevations. The sub-arctic climate has low precipitation (300–400 mm annually with ∼50% falling as rain), cool summers (monthly mean temperatures of 5 to 15°C for June–August) and cold winters (monthly mean temperatures of −10 to −20°C for December–February) [31].

**Figure 1:**
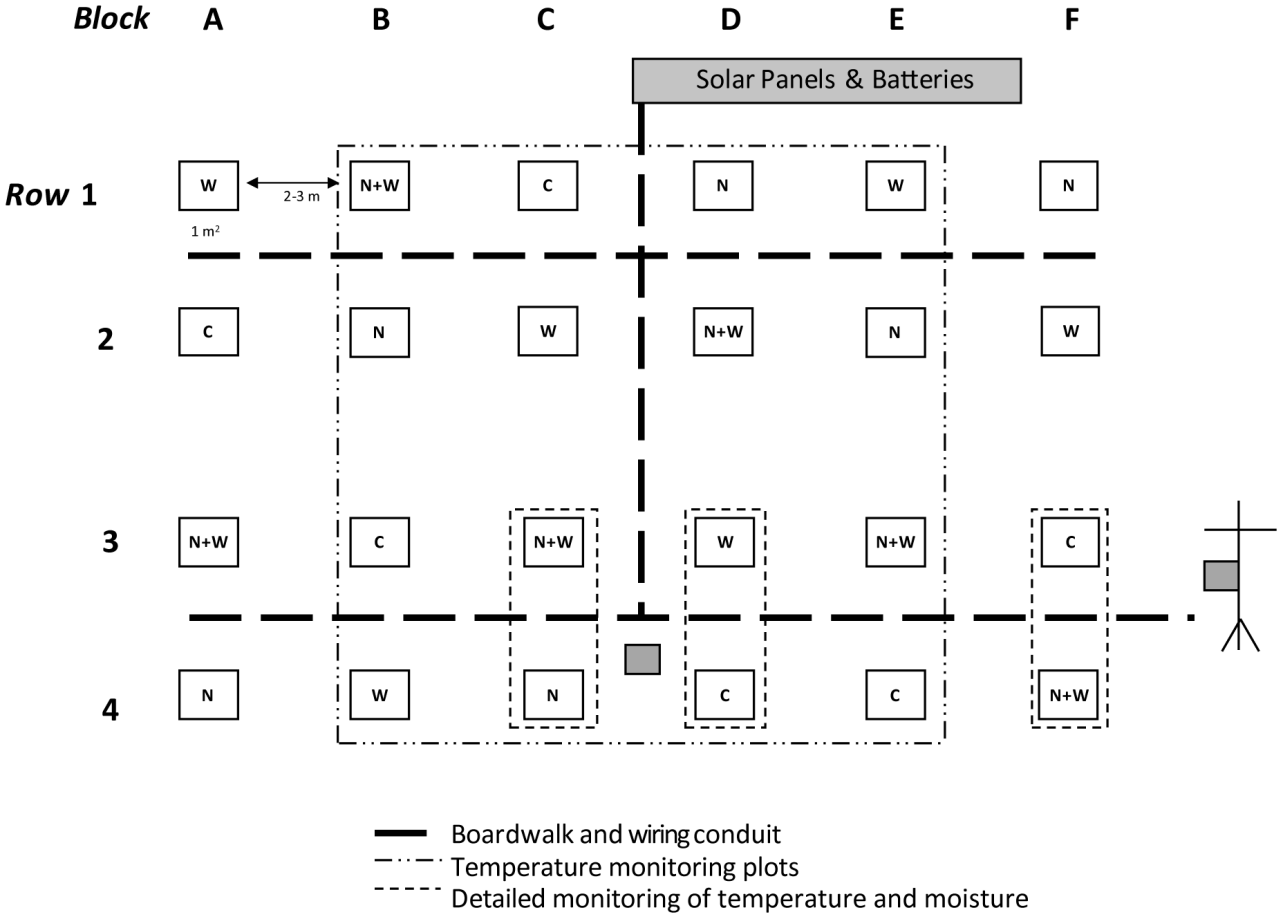
Site map showing the layout of 24 experimental plots in a randomized block design. Plots were randomly assigned to two crossed experimental factors, warming (W), nitrogen fertilization (N) and associated ambient controls (C). As of 2012, the low-dosage N treatment had no observable effects on vegetation is not addressed in this paper. Experimental plots (1 m^2^) were connected to the warming system via wires encased in conduit (thick dashed lines). Grey-filled boxes indicate the location of three datalogger units, including one associated with the solar power supply and a second located on the micro-meteorology tower. The large dashed-dotted box encloses the 16 plots that were monitored for temperature by the control system; smaller dashed lines indicate 6 plots with more detailed monitoring of temperature profiles and soil moisture. Note the drawing is not to scale.

Experimental plots were established in a 30×50 m patch of visually homogeneous tundra vegetation located near the top of a gently sloping (∼3°), south-facing ridge (Fig. 1). Soils in the area are Orthic Eutric Brunisols [32] and have a texture that is predominantly sandy loam with approximately 50% (by volume) irregularly shaped coarse gravel and cobble fragments. Although the soils show evidence of cryoturbation in the form of broken horizons, downward migration of surface organic material (Om and Ah horizons), and earth hummocks, permafrost was not observed within the upper 2 m of soil. A discontinuous organic layer (Om horizon) of up to 4 cm depth overlays the surface of the mineral soil. Total cover of vascular plants at the site is about 40% and is dominated by prostrate woody shrubs (*Dryas octopetala* and *Salix* spp.) [25]. Lichens and scattered patches of moss cover much of the remaining soil surface. No protected species were disturbed by this research.

### Design Specifications

**Figure 2:**
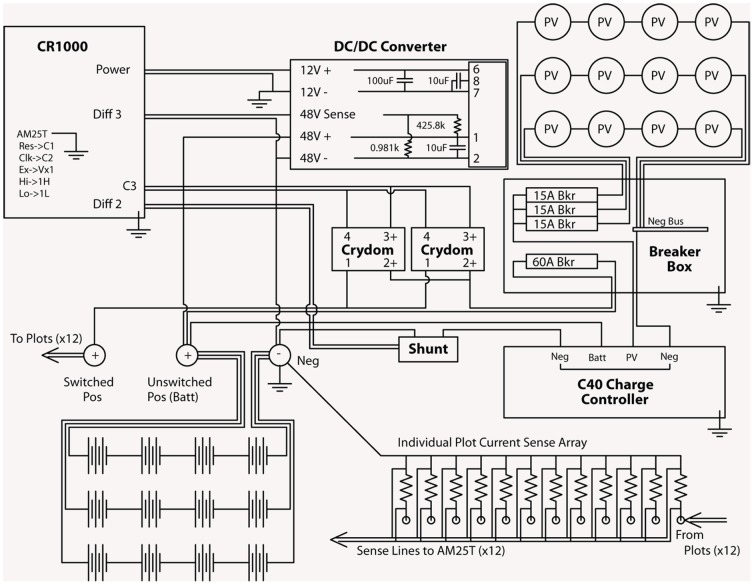
Circuit diagram of the experimental control system. The system is monitored and controlled by a central datalogger (CR1000) that receives information on plot temperatures and power output via multiplexer (AM25T) connected to thermocouples and electrical shunts for 12 experimental plots (connections 1–24 on the AM25T). An array of photovoltaic panels (PV) is connected to electrical breakers and a charge controller to provide power to a 48-volt battery bank. Solid-state relays are used by the datalogger to turn power on and off to the experimental plots. The following abbreviations are used in the diagram: batt = battery, bkr = breaker, diff = differential circuit, neg = negative, pos = positive, pv = photovoltaic.

The experimental warming system was designed to provide controlled warming of near-surface soils during the summer snow-free period, without connection to grid power. The system consisted of three sub-components: a power supply, a heating system, and a monitoring and control system (Fig. 2). The power supply used a photovoltaic array to charge a deep-cycle battery bank that provided a regulated source of diurnal power. Twelve, 115 W photovoltaic panels (BP Solar, Model BP3115J) provided power to an array of twelve 12 V, 114 Ah deep cycle batteries (Discover, Model EV31A-A) via a charge controller. Three sets of four 12 V batteries were wired in series to obtain a 48 V power system. Increasing the voltage to 48 V generated a lower current system that was safer to use, reduced energy loss in the wires by a factor of 16, and made current-carrying components less expensive and easier to obtain. A 48V to 12V DC/DC converter was used to provide power to system components that required 12 V power.

The heating system converted electricity to heat in the ground using a network of heating probes constructed from 20 cm lengths of resistive heating wire (nickel-chromium, 22 gauge, 3.5 Ω m^−1^, Omega Engineering Inc, Stamford, CT, USA). The wire was doubled back on itself to form a 10 cm loop, which was then insulated and sheathed with adhesive shrinkable tubing. The electrical design for each plot consisted of 100 probes constructed as two parallel circuits of 50 probes connected in series. Individual probes had a resistance of 0.7 Ω and thus the expected rate of heating for each plot was 132 W. Standard electric wire (i.e. non-heating) was used to connect the probes to each other (16 AWG) and back to the power supply (14 AWG). Soldered joints were waterproofed with adhesive heat-shrink tubing, and exposed wires were encased in conduit or braided metal sheathing to protect them from being chewed on by animals.

To minimize disturbance to vegetation and soil, heating probes were installed vertically in the soil. Each warming plot had 100 heating probes that were equally spaced in a 10 cm×10 cm grid within the 100 cm×100 cm plot. We created pilot holes for installing the probes by pounding a 3 mm diameter metal rod 15 cm into the ground. The 10 cm long probes were positioned in the holes so that the heating length was 5–15 cm below the soil surface. Rocks in the soil were accommodated by small variations in probe location or orientation.

**Figure 3:**
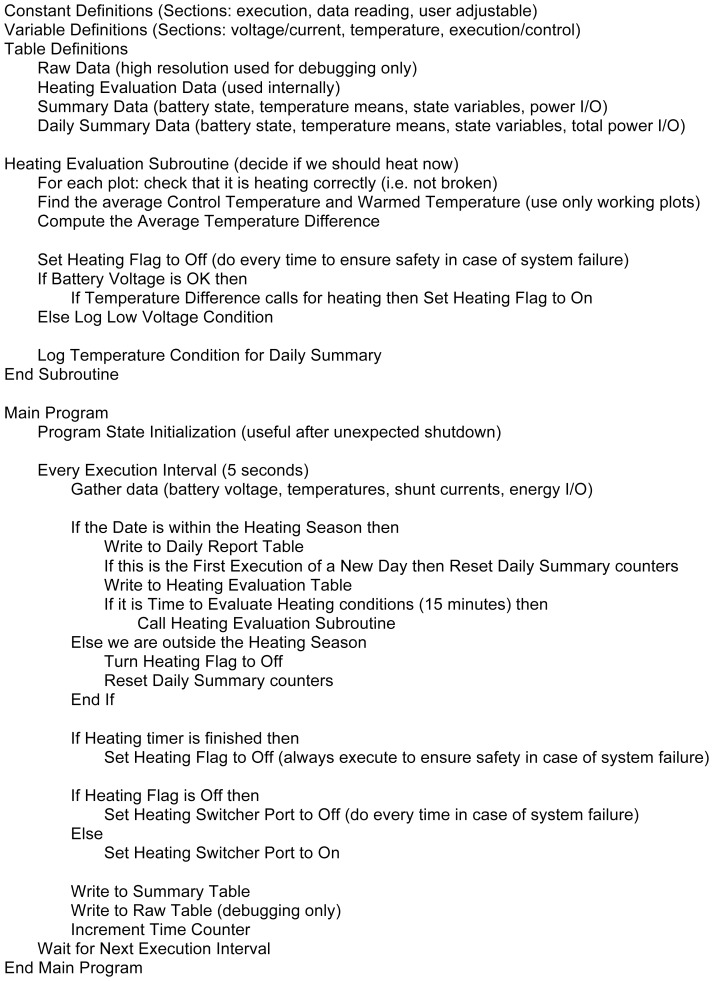
A pseudocode outline of the algorithm used to monitor effects and control the heat output of the warming system.

The monitoring and control system used a CR1000 (Campbell Scientific, Edmonton, AB, Canada) programmable datalogger to control heat output and target a set level of warming. The datalogger monitored battery bank voltage (V), charge current (A), and soil temperatures (°C) in warming and control plots (n = 6/treatment), as well as the energy output to each plot. These parameters were evaluated over a specified time period (e.g. 15 minutes), referred to here as a “heating cycle”, and the datalogger then switched on/off the heating circuitry based upon the programmed control logic. Control logic ensured that heating extremes were avoided and minimized the risk of over-discharging the battery bank (Fig. 3).

The amount of energy delivered to the plots was set by adjustments to the duty cycle, i.e, the portion of a heating cycle in which power was switched on. In this experiment, the heating cycle was set to 15 minutes and the duty cycle was ∼2 minutes. The datalogger used shunt resistors to determine the energy (kJ) supplied to the experimental plots (Fig. 2). The control system was mounted on a plywood panel to provide easy access for maintenance and debugging (Fig. S1). This circuit board and the battery bank were enclosed in sealed plywood boxes to protect the electrical components from weather and rodent damage.

We developed control code for the CR1000 datalogger using the CR-Basic programming language (Campbell Scientific, Edmonton, AB, Canada). Control code (Fig. 3) was aimed at balancing the following objectives: (a) a minimized the risk of damage to system components (especially the battery bank) while (b) achieving the target warming level with (c) an even heat output over time; (d) ensuring the system defaulted to a safe operation mode in the event of failing components; (e) developing a code structure that was easy to understand, upgrade, and maintain, and (f) functioned optimally at the wide range of environmental conditions dictated by the field situation (e.g. sunny vs. cloudy weather). As there is no single definition of “optimal” system operation, we chose to prioritize our list in the order given above. Optimizing battery health was complicated by the fact that the battery bank rarely reached a full charge because of the continuous power draw from the warming system. Hence, we used a voltage-based approach to determine state of charge, thereby avoiding problems of long-term drift in our state of charge calculations. Figure 3 provides a pseudocode outline of our control algorithm, and a full code listing is available in Fig. S2.

### Field Measurements

The experimental warming system was installed in June–July 2010. Initial testing occurred in late July and early August of 2010, and the system was run for a full growing season from 24 May to 31 August, 2011. Experimental plots were laid out in a randomized block design with 2 crossed experimental treatments, warming and a low dosage nitrogen fertilization treatment (2 g N m^−2^) (Fig. 1). The fertilization treatment was initiated in 2011 and had no observable effects on vegetation or soils in 2011 or 2012 (Johnstone, unpublished data), so is not considered further here. Field measurements and analyses focused on the 12 replicates of the warming treatment (warming and control). Plots were placed 2.5–3.0 m apart to achieve a compact physical layout that minimized power loss across electrical distribution wires but still allowed a reasonable buffer space between plots (Fig. 1).

We monitored soil temperatures with type “T” thermocouples (copper-constantan, 24 gauge, Omega Engineering Inc., Stamford, CT, USA) placed at a depth of 10 cm below the soil surface unless otherwise noted. Temperature measurements used by the control system were based on 3 electrically averaged thermocouple leads positioned 8–15 cm from each other in 6 warming and 6 control plots. We also installed supplementary arrays of thermocouples to characterize spatial variations in soil temperature in three randomly selected pairs of warming and control plots. We monitored horizontal temperature profiles using 5 thermocouples positioned at a uniform depth of 10 cm and horizontal distances of 1, 4, 7, 10, and 13 cm along a 14 cm transect, equal to the diagonal distance between two heating probes. For vertical profiles, we mounted thermocouples on a thin wooden dowel and inserted them in a vertical pilot hole to record temperatures at exponentially increasing depths of 3, 6, 12, 24, and 48 cm below the soil surface.

We monitored changes in volumetric soil moisture using single time-domain reflectometry probes (CS616 Water Content Reflectometer, Campbell Scientific, Edmonton, AB, Canada) inserted in the upper 20 cm of soil in each plot (n = 6). Measurements of temperature and moisture were recorded at 15-minute intervals.

Statistical analyses used plots as the unit of replication. We dealt with the non-independence of repeated measures through time by either explicitly modeling a 1^st^ order autocorrelation structure in the data (used to test treatment effects on daily minimum, mean, and maximum temperatures), or by averaging values from thermocouples within plots to obtain a single value (used for temperature profile data). Where multiple temperature sensors were nested within plots, we accounted for the spatial dependence using mixed effects models [33]. Treatment effects on temperature variation were assessed with one-way ANOVA with the standard deviation of temperature observations as the response variable. Analyses were performed using the software program R v.2.15.2 [34].

## Results

**Figure 4:**
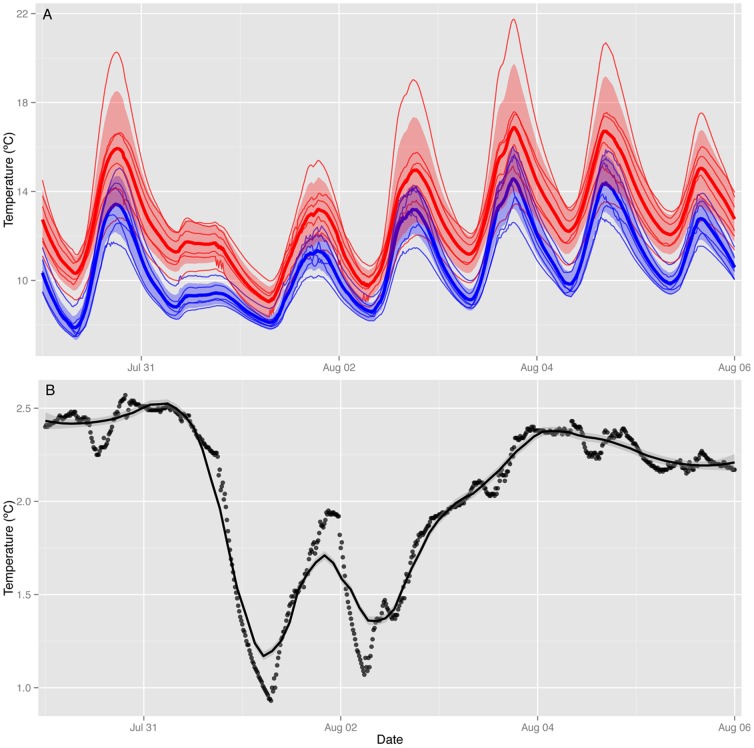
Summary of the warming treatment effect on observed soil temperatures from midnight of 30 July to midnight of 6 August 2010. Average soil temperatures in (A) are shown as thick lines for warmed (red, n = 6) or control (blue, n = 6) treatments, with shaded areas indicating one standard deviation around the mean. Treatment effects are summarized in (B) as the mean temperature difference between warming and controls. Temperatures were recorded at 10 cm depth every 15 minutes from 0:00 to 24:00 and represent an average across three temperature sensors per plot.

Initial trials during the 7-day test period in 2010 demonstrated that the warming system was capable of significantly increasing daily mean soil temperatures to the target warming objective of 2°C (mean temperature increase of 2.1°C±0.6 SE; t = 3.41, p = 0.007). Diurnal patterns of soil temperature in the warming plots closely tracked patterns observed in the controls (Fig. 4a). Most importantly, lines representing mean temperatures in warming and control plots never crossed, indicating that the system maintained a positive warming differential at all times (Fig. 4b). The heating system maintained a relatively constant increase of ∼2°C during the initial test period except during cloudy periods, when power reserves became limited. Because incoming solar power was centered around mid-day, power shortages were typically realized at night, leading to reduced night-time warming (Fig. 4b). Active control of the soil warming system prevented overheating and unusual temperature extremes, so that the warming effects on daily minimum and maximum temperatures were similar to each other (means ± SE of 2.0±0.5°C and 2.1±1.0°C for minimum and maximum temperatures, respectively).

**Figure 5:**
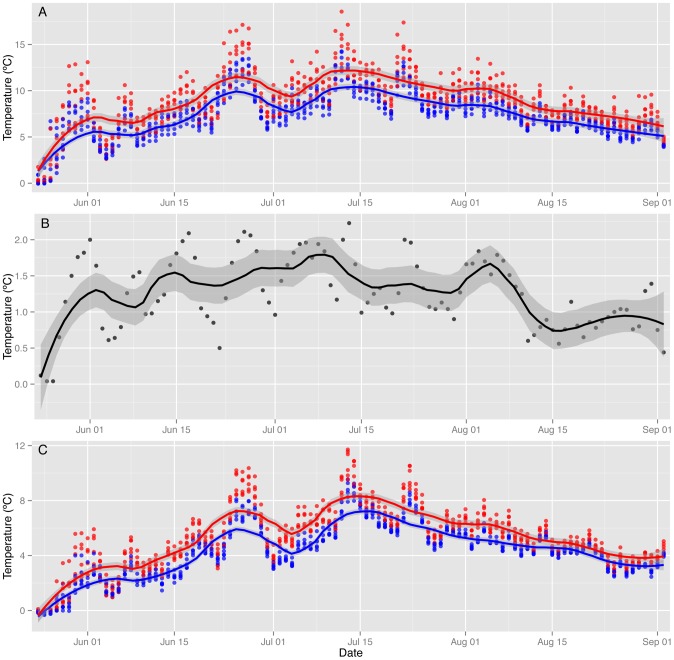
Daily soil temperatures recorded in control plots (blue dots, n = 6) and warmed plots (red dots, n = 6) over a complete warming season from 24 May to 1 September 2011. Temperatures are summarized as (A) daily maximum temperature, (B) the difference in daily mean temperatures between warming and control plots, and (C) daily minimum temperature. In each panel, treatment means are shown as a solid line (red = warmed, blue = control, black = temperature difference) with one standard deviation indicated by grey shading.

During the longer test period in 2011, soil heating started on 24 May and reached the target differential of 2°C within one week (Fig. 5b). However, heating performance varied through the season. Days with cloudy weather reduced solar energy input and constrained the heating capacity of the system, especially in late summer (Fig. 5). As a result, the treatment effect on mean daily temperatures over the full 2011 test period was 1.38±0.51°C (t = 2.706, p = 0.007). The system maintained a positive temperature differential between warming and control plots throughout the season (Fig. 5b), with consistent effects on daily maximum (mean 1.62±0.47°C, t = 3.448, p<0.001; Fig. 5a) and minimum temperatures (mean 1.04±0.44°C, t = 2.368, p = 0.018; Fig. 5c).

**Figure 6:**
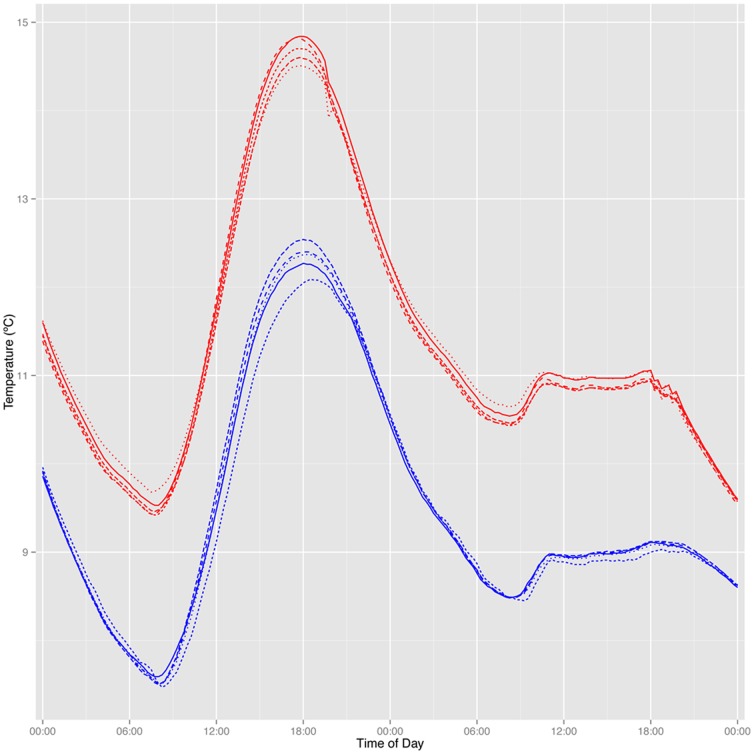
Diurnal patterns of temperature observed across horizontal profiles in warmed (red, n = 3) and control (blue, n = 3) plots over a 48 hour period that included a sunny and cloudy day on July 30–31, 2010. Thermocouples recorded soil temperatures at a depth of 10

Temperature sensors distributed along horizontal transects within the plots showed similar patterns of spatial variability in soil temperature between treated and control plots. For example, the difference in temperatures recorded 1 cm vs. 7 cm away from a heating probe (the maximum possible distance) was similar to the natural level of soil temperature variation seen across similar distances in the control plots (Fig. 6). We found no significant differences in horizontal temperature variation (measured as within-plot standard deviations) between treatments (F = 0.06, p = 0.8 for the test period in 2010 and F = 0.28, p = 0.6 over the same period in 2011). Temperature variations in the control plots are attributable to natural, microscale variations in soil physical properties and vegetation cover that affect soil temperatures. Our data suggest that any horizontal gradients in temperature introduced by the heating probes lie within the range of this natural variability.

**Figure 7:**
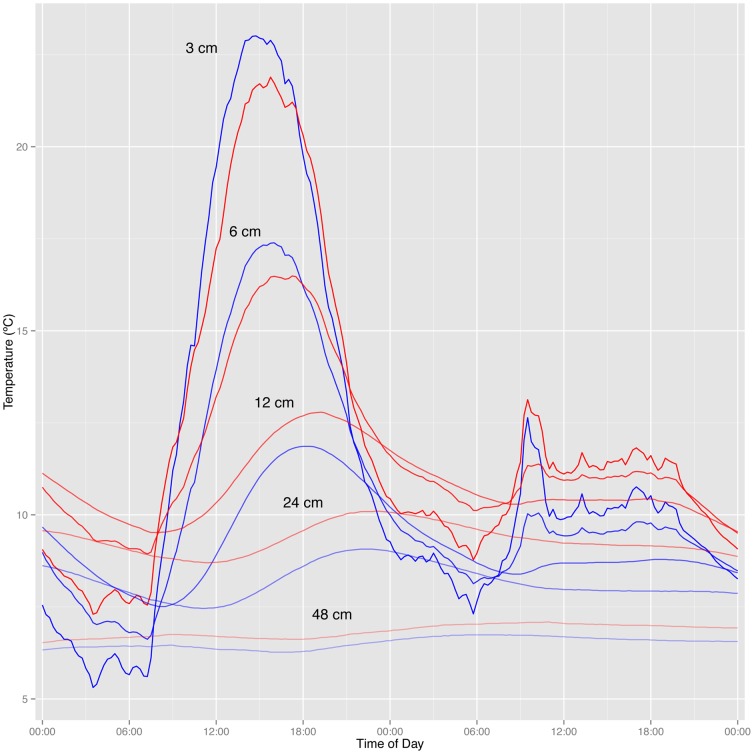
Diurnal patterns of temperature observed across vertical profiles, averaged across three warmed plots (red) and three control plots (blue). For illustration purposes, temperatures are shown over a 48–31, 2010. Thermocouples recorded soil temperatures at depths of 3, 6, 12, 24, and 48 cm below the soil surface. During the warmest portion of the record (midday on July 30), the ordering of the lines directly reflects the vertical position of the probes, with thermocouples closest to the soil surface showing the warmest temperatures and the deepest probes showing the coldest temperatures (color saturation of the lines decreases with depth).

Vertical profiles of soil temperature in the 6 plots with detailed data suggested variable effects of the warming treatment with depth. As expected, vertical position had the strongest effect on observed mean soil temperatures (p<0.01 for all periods). Temperature patterns in both sets of plots were strongly influenced by diurnal cycles, which became weaker in amplitude with depth (Fig. 7). Natural variation among plots led to some of the warmed plots having cooler near-surface temperatures than the controls, likely associated with patterns of vegetation shading. As a consequence of the high variability among plots, we saw no significant effects of warming on mean temperatures across the vertical profiles in the first half of the 2011 season (mixed effects model, t = 1.00, p = 0.37 for June 1 to July 15). However, there was some indication of a significant warming effect through the soil profile in the later part of the 2011 season (mixed effects model, t = 2.6, p = 0.02 for June 15 to August 31). There were no significant interactions between depth and warming in either early or late summer (p = 0.70 and 0.17, respectively). These data suggest that warming effects likely extend through a large volume of soil but require several weeks become strong enough to be detectable above the background variability in temperatures among plots.

**Figure 8:**
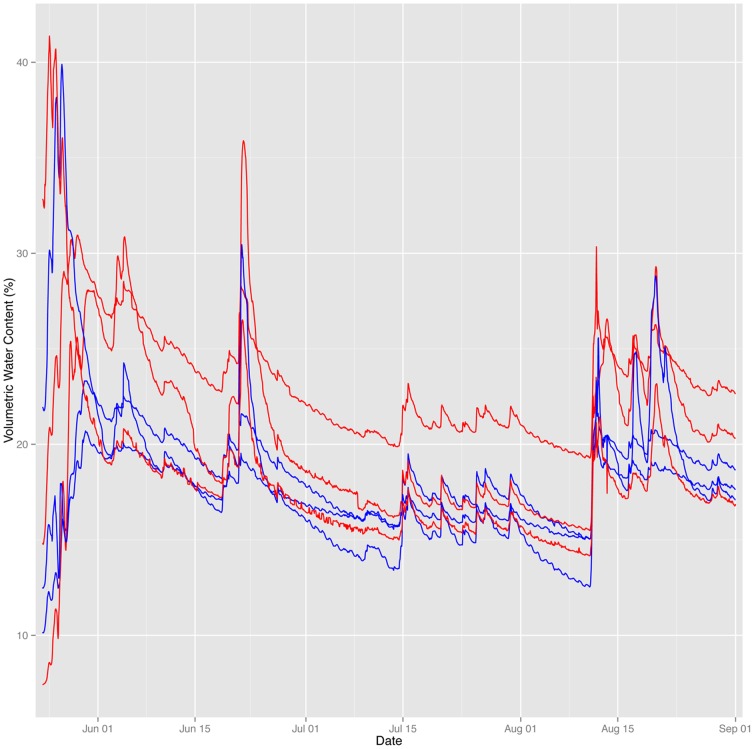
Relative change in soil volumetric moisture content (%) observed in warmed (red lines; n = 3) and control (blue lines; n = 3) plots recorded over a complete warming season from 24 May to 1 September 2011.

Changes in soil temperature may affect soil moisture by altering rates of water evaporation from the soil. In 2011, soil moisture at the study site peaked at the start of the growing season following snow melt, and was followed by a gradual drying that was interrupted occasionally by precipitation events (Fig. 8). Similar patterns were seen during the shorter test period in 2010. We observed high variability in soil moisture between plots with no consistent pattern between warming and control treatment, indicating that small-scale variations in topography and soil drainage had the dominant effect on soil moisture patterns. By early July, 5 of 6 plots showed very similar levels of soil moisture, while 1 warmed plot that was located in a small depression remained consistently wetter than the others throughout the summer (Fig. 8).

## Discussion

Our field trials indicate that a system of electrical heating probes tied to a remote power supply and control system can achieve a stable warming of 1–2°C in near-surface soils. This is the first report of an active warming system that does not rely on a source of grid power, and as such, it opens up the possibility of a new generation of active warming experiments for remote regions. Active, automated control of the warming system resulted in a consistent warming treatment that avoided extreme effects on minimum and maximum temperatures with minimal operational inputs. Overall, the power requirements for this system were low compared to other designs for active temperature manipulation in the field [11], [27], [28], [29]. Power use in this design was also highly efficient as virtually all of the energy used (excepting only line losses) was directly transferred to heat in the soil.

A key feature of the warming treatment tested here was the ability to increase soil temperatures without substantially altering natural patterns of temperature variability. This can likely be attributed to the use of a short duty cycle, where heat was applied for relatively short intervals (e.g. ∼2–3 minutes) during a heating cycle (15 minutes). These brief heating pulses were not long enough to allow extreme temperature gradients to develop around the heating probes. Detailed spatial measurements suggest that heat energy rapidly dissipated through the soil via conduction resulting in relatively even heating of the soil volume. Natural variations in near-surface temperatures at the scale of a few cm were of a similar magnitude to the temperature gradients observed around the warming probes. We also found that concentrating heat input in the near-surface soil maintained vertical temperature gradients similar to those in control plots, while allowing temperature effects to propagate to depths well below the heating zone. Measurements of soil moisture suggest that, although the manipulation resulted in significantly warmer soil temperatures, these changes did not substantially alter soil moisture beyond the level of background variation. Together, these results suggest that the direct warming of near-surface soil with vertical heating probes can successfully alter soil temperatures while maintaining realistic spatial temperature gradients and moisture levels in the soil. This is important because it allows any treatment effects on biotic processes to be more confidently attributed to warming, rather than to secondary physical effects such as soil moisture, or localized high temperatures around the heating probes.

The use of vertical heating probes instead of horizontal heating cables [12], [28], [30], caused little disturbance to surface vegetation and easily accommodated rocky, gravelly soils and variations in organic horizon depth or surface cover. The small pilot holes used for probe installation allowed the vegetation and surface organics to remain intact and largely untouched, and the exact position of a probe could be modified to leave rocks and other obstructions in place. Thus, vegetation and soil responses to the warming treatment are unlikely to be confounded by strong disturbance impacts that could otherwise overwhelm subtle warming effects [15]. Furthermore, the amount of equipment installed in or around individual plots was minimal, being confined largely to conduit and short loops of wires. Minimizing equipment installations around experimental plots may reduce the tendency of warming manipulations to act as a barrier to herbivores and thereby confound climate manipulations with altered herbivory [35], [36].

The control portion of the system incorporates several flexible design elements that allow the system to be customized to the specifics of a site. For example, because the thermal conductivity of soil depends on moisture and texture, specification of the most effective heating cycle length for even soil warming is likely to vary. Our system specifically allows for heat inputs to be fine-tuned to soil conditions by allowing the user to specify the length of the heating cycle (frequency of heat input) separately from the duty cycle (proportional duration of heat input). The system is also flexible in allowing the user to fit warming performance to the amount of power available. Thus, even under weather conditions that may limit power availability from photovoltaics or wind turbines, the system will attempt to achieve some level of warming. Our trials indicate that uniform warming over time is likely to be very difficult when energy is limited. However, the flexible control of this system should minimize the high level of site-specificity and annual variability in warming that is characteristic of passive warming designs [15], [24]. Finally, flexibility within the control system allows a variety of warming designs to be implemented, such as targeted early or late-season warming [37], day or night time warming [11], or field manipulation of soil freeze-thaw cycles [38].

Perhaps the greatest potential of the system design presented here is its ability to achieve a target level of controlled soil warming without access to a large supply of electrical power. However, there are limitations to the amount of power that can be supplied from a given alternate energy source. For example, we observed declines in heating performance during extended cloudy weather or in late summer when solar input was reduced. Our application was intended to only operate during the summer growing season, and so reliance on solar power as a primary energy source seems appropriate; clearly, winter applications at high latitudes would require a different source of power. In theory, the system can be scaled to warm larger volumes of soil by increasing the size and number of heating probes. In practice, the amount of soil that can be warmed will depend on the power available, and substantial increases in the scale of the experiment will require tapping into additional sources of power. Power limitations observed within our experimental trials could likely be best addressed by adding an alternate power source, such as a wind turbine, that can supply power during periods of cloudy weather and in late summer. Constraints on the type of warming achieved (i.e. only soil warming) may also be addressed by combining this system with other manipulations. For example, passive warming techniques such as open-topped chambers (OTCs) are typically able to significantly warm air temperatures but often have less consistent effects on soil temperatures [18], [20], [21], [24]. A hybrid system that uses the soil warming system described here in combination with OTCs could provide a new model for ecosystem-level climate manipulations that would address potential weakness of both designs (e.g. [30]).

The results of this study show that controlled warming with vertical heating probes is a useful approach to experimental warming of field soils, and that powering such systems by alternative energy is viable for remote locations. Electrical heating probes installed vertically in the soil minimize surface disturbance while maintaining realistic soil temperature profiles and adaptability to a variety of soil conditions. This soil warming system should be easy to adapt to other power sources, scale to different spatial designs, or use in combination with other environmental manipulations. Given the central role of soil processes in constraining ecosystem responses to climate change, we hope that this design will facilitate new experimental work on the ecological impacts of soil warming, particularly in under-studied, remote parts of the globe.

## Supporting Information

Figure S1 **An annotated photo of the control board used in the field installation.** Major system components are labeled with text and arrows (see the main text for details on individual system components). Note the upper image has been spliced to remove a support strut that concealed part of the control board.
(TIF)

Figure S2 **Actual code used for running the CR1000 datalogger for system monitoring and control (executable code in black font, non-executable comments in blue).**
(PDF)
