# Supplementary figures and images for: Controlled Soil Warming Powered by Alternative Energy for Remote Field Sites
Source: PLoS One. 2013 Dec 26;8(12):e82903. doi: 10.1371/journal.pone.0082903 (PMC3873302; doi:10.1371/journal.pone.0082903)

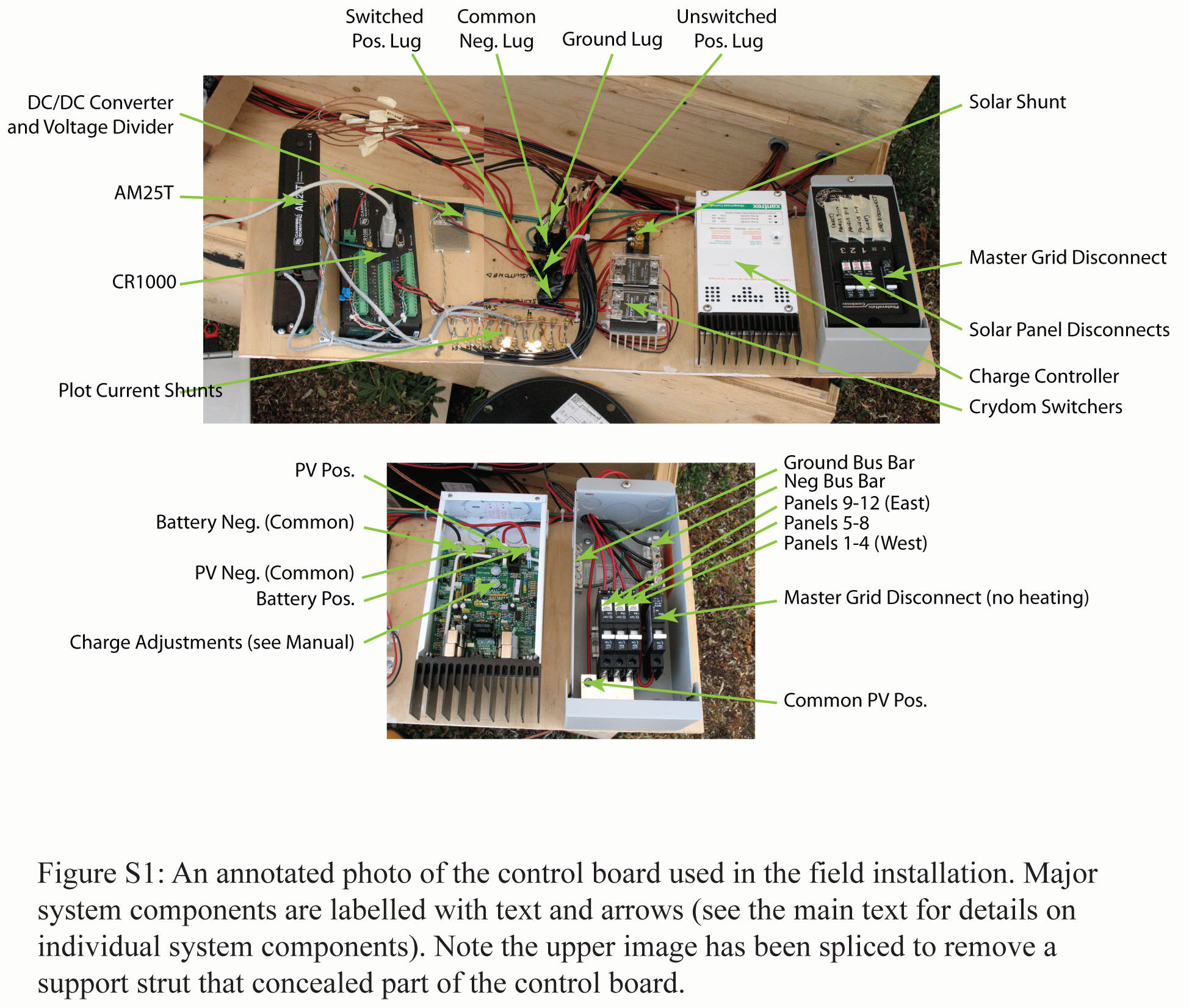

Supplement: Figure S1 — An annotated photo of the control board used in the field installation. Major system components are labeled with text and arrows (see the main text for details on individual system components). Note the upper image has been spliced to remove a support strut that concealed part of the control board. (TIF) [file pone.0082903.s001.tif]
