# Supplementary material for: Controlled Soil Warming Powered by Alternative Energy for Remote Field Sites
Source: PLoS One. 2013 Dec 26;8(12):e82903. doi: 10.1371/journal.pone.0082903 (PMC3873302; doi:10.1371/journal.pone.0082903)
Supplement: Figure S2 — Actual code used for running the CR1000 datalogger for system monitoring and control (executable code in black font, non-executable comments in blue). (PDF) [file pone.0082903.s002.pdf]

Table S2: Actual code used for running the CR1000 datalogger for system monitoring and control. Executable code is shown in black font; non-executable comments are in blue font.

```
'CR1000 Series Datalogger
'$$$$$$$$$$$$$$$$$$$$$$$$$$$$$$$$$$$$$$$$$$$$$$$$$$$$$$$$$$$$$$$$$$$$$$
' Declare CONSTANTS

' Execution Constants - redefinition of these implies reallocating data tables
Const START_DAY = 144          ' Jday to begin warming of plots [May 24]
Const END_DAY = 244            ' Jday to end warming of plots [Sept 1]
Const OPTIMIZATION_TIME = 6    ' Clock time (in hours) of new day; so charging doesn't influence heating
Const EXEC_INTERVAL = 5        ' Execution interval in seconds -- MUST BE EVENLY DIVISIBLE INTO 1 MINUTE
Const EVAL_INTERVAL =  15*60   ' Period in seconds over which to evaluate temperatures and summary data
Const EVAL_AVERAGING = 60      ' Time (sec) to avg data (end of EVAL_INTERVAL) for deciding to heat

' Data reading constants
Const VOLT_DIVIDER = 0.4442405708 ' Convert from mV to 48V to check the main battery voltage
Const R_SHUNT = 0.01           ' Resistance of shunt resistors for warming plots; converts from mV to Amps
Const R_SHUNT_SOLAR = 0.001    ' Resistance of solar charge shunt; comverts from mV to Amps

ConstTable
' Duty cycle constants
Const DUTY_CYCLE_DEFAULT = 0.17 ' Default duty cycle as decimal fraction (initial value at boot time, but
see PreserveVariables call); empirically determined that 17% is minimum to get through the day at adequate
heat.

' Heating and optimization constants
Const SWITCHER_PORT = 3        ' Location of Crydom switcher trigger
Const BATT_V_MIN = 12.0 * 4    ' If voltage falls below this value do not heat and log a volt too low fault
Const T_DELTA_MAX = 2.5        ' Heat plots if temperature difference is below this value
Const T_DELTA_MIN = 1.8        ' Log a low temperature fault if difference is below this value
Const VALID_HEAT = 0.7         ' Less heating than this and we assume the plot is broken
EndConstTable

'$$$$$$$$$$$$$$$$$$$$$$$$$$$$$$$$$$$$$$$$$$$$$$$$$$$$$$$$$$$$$$$$$$$$$$
' Declare Variables
```

```
PreserveVariables

' Voltage/Current variables
Public V_logger As Float              ' voltage of logger battery
Public V_battery As Float             ' voltage of battery array
Public A_shunt(12) As Float           ' current across each individual plot shunt
Public A_solar As Float               ' current across solar charge shunt
Public kJ_shunt(12) As Float          ' energy (kJ) delivered to individual plots
Public kJ_solar As Float              ' energy transferred (kJ) by solar supply shunt

' Temperature variables
Public T_panel As Float               ' panel temperature of the CR1000
Public T_controls(12) As Float        ' array containing all 12 control temperature measurements
Public T_cont_avg As Float            ' the spatial averaged temperature of the non-warmed plots
Public T_warm_avg As Float            ' the spatial averaged temperature of the warmed plots

' Execution variables and counters
Public Duty_Cycle As Float            ' the duty cycle used in the program
Public Duty_Counter As Long           ' maintains the execution point (CR1000 cannot > or < a time value)
Public Heat_Flag As Boolean           ' main heating request flag
Dim Process_Disable As Boolean        ' disable intermediate processing of temp / voltage during heating
Public Temp2low As Long               ' counts occurrences when the temp difference falls below T_DELTA_MIN
Public Temp2high As Long              ' counts occurrences when the temp difference is above T_DELTA_MAX
Public Volt2low As Long               ' counts occurrences when the battery voltage falls below BATT_V_MIN
Dim I As Long                         ' a general purpose loop control variables

'$$$$$$$$$$$$$$$$$$$$$$$$$$$$$$$$$$$$$$$$$$$$$$$$$$$$$$$$$$$$$$$$$$$$$$
' Aliases - Note these reflect wiring order

' Total shunt energies
Alias kJ_shunt(1) = kJ_A1_W
Alias kJ_shunt(2) = kJ_A3_NW
Alias kJ_shunt(3) = kJ_B4_W           ' Note B1 and B4 are reversed because we need the data contiguous for
Heat_Eval subroutine
Alias kJ_shunt(4) = kJ_B1_NW          ' See previous note
Alias kJ_shunt(5) = kJ_C2_W
Alias kJ_shunt(6) = kJ_C3_NW
Alias kJ_shunt(7) = kJ_D2_NW
Alias kJ_shunt(8) = kJ_D3_W
```

```
Alias kJ_shunt(9) = kJ_E1_W
Alias kJ_shunt(10) = kJ_E3_NW
Alias kJ_shunt(11) = kJ_F2_W
Alias kJ_shunt(12) = kJ_F4_NW

' Control Temperatures
Alias T_controls(1) = T_B2_N
Alias T_controls(2) = T_C1_C
Alias T_controls(3) = T_C4_N
Alias T_controls(4) = T_D1_N
Alias T_controls(5) = T_D4_C
Alias T_controls(6) = T_E2_N
Alias T_controls(7) = T_B1_NW
Alias T_controls(8) = T_C2_W
Alias T_controls(9) = T_C3_NW
Alias T_controls(10) = T_D2_NW
Alias T_controls(11) = T_D3_W
Alias T_controls(12) = T_E1_W

'$$$$$$$$$$$$$$$$$$$$$$$$$$$$$$$$$$$$$$$$$$$$$$$$$$$$$$$$$$$$$$$$$$$$$$$
' Units

Units V_logger = Volts
Units V_battery = Volts
Units A_shunt = Amps
Units A_solar = Amps
Units kJ_shunt = kJ
Units kJ_solar = kJ
Units T_panel = *C
Units T_controls = *C
Units T_cont_avg = *C
Units T_warm_avg = *C
Units Duty_Cycle = %

'$$$$$$$$$$$$$$$$$$$$$$$$$$$$$$$$$$$$$$$$$$$$$$$$$$$$$$$$$$$$$$$$$$$$$$$
' Define Data Tables
'TABLE: Raw data for debugging. In winter this table needs to be COMMENTED OUT to give enough memory
DataTable (Raw,True,48*3600/EXEC_INTERVAL)      ' Store 48H of data so as not to compromise logger storage
      DataInterval (0,EXEC_INTERVAL,Sec,-1)
```

```
      Sample (1,V_logger,FP2)        ' Logger battery voltage: output from the 12V DC-DC converter
      Sample (1,V_battery,FP2)       ' Voltage of the 48V battery bank
      Sample (12,T_controls(),FP2)   ' Temperature for each plot used for control (6 warmed, 6 control)
      Sample (12,A_shunt(),FP2)      ' Actual reading from the shunt resistor
      Sample (1,A_solar,FP2)         ' Actual reading from the solar shunt.
EndTable

' TABLE: Measurement of controlling variables over the last minute of EVAL_INTERVAL for heating decision
making purposes.
DataTable (Heat_Eval,True,2*(END_DAY-START_DAY)*24*3600/EVAL_INTERVAL)  ' CR1000 cannot know this will not
be called all winter so we need to do it manually (2 seasons worth)
      DataInterval (0,EVAL_INTERVAL,Sec,10)           ' Give 10 lapses; should compensate for the winter
      Average (1,V_battery,FP2,Process_Disable)       ' Voltage of the 48V battery bank
      Average (12,T_controls,FP2,Process_Disable)     ' Temps of plots being monitored by the control sys.
      Totalize (12,kJ_shunt(),FP2,False)              ' Total energy to each plot over last EVAL_INTERVAL.
EndTable

'TABLE: Summary data: Output data useful for postprocessing and debugging at the evaluation interval level.
This is the primary output table.
DataTable (Summary,True,-1)                 ' Use the remaining memory here: should yield about a 1 1/2 years
      DataInterval (0,EVAL_INTERVAL,Sec,10)
      Average (1,V_logger,FP2,False)        ' Logger battery voltage: actually the from the DC-DC converter
      Average (1,V_battery,FP2,False)       ' Voltage of the 48V battery bank
      Average (1,T_panel,FP2,False)         ' Panel temperature
      Average (12,T_controls(),FP2,False)   ' Temperature for each plot used for control (6 warm, 6 control)
      Sample (1, T_cont_avg,FP2)            ' Computed avg temperature for the unheated plots in this eval.
      Sample (1, T_warm_avg,FP2)            ' Computed avg temperature for the heated plots in this eval.
      Sample (1,Heat_Flag,UINT2)            ' Indicates if we will heat in the upcoming cycle.
      Totalize (12,kJ_shunt(),FP2,False)    ' Total energy to each plot in the last EVAL_INTERVAL.
      Totalize (1,kJ_solar,IEEE4,False)     ' Total energy form the solar panels in the last EVAL_INTERVAL
EndTable

'TABLE: Daily Summary: Output data that summarizes the daily configuration of the system and useful data
for debugging optimization.
DataTable (Daily_Report,True,2*(END_DAY-START_DAY))   ' CR1000 cannot know this will not be called all
winter so we need to do it manually (2 seasons worth)
      DataInterval (OPTIMIZATION_TIME,24,Hr,0)
      Average (1,V_battery,FP2,False)       ' Average battery voltage over the day
      Average (1,T_cont_avg,FP2,False)      ' Daily average temperatures in the control plots
```

```
      Average (1,T_warm_avg,FP2,False)     ' Daily average temperatures in the warming plots
      Totalize (12,kJ_shunt(),FP2,False)   ' Total power sent to each plot
      Totalize (1,kJ_solar,IEEE4,False)    ' Total power gathered by the solar panels
      Sample (1,Volt2low,UINT2)            ' Number of low volt events – for reference only
      Sample (1,Temp2low,UINT2)            ' Number of low temp events - for reference only
      Sample (1,Temp2high,UINT2)           ' Number of high temp events - for reference only
      Sample (1,Duty_Cycle,FP2)            ' Store the current duty cycle
EndTable

'$$$$$$$$$$$$$$$$$$$$$$$$$$$$$$$$$$$$$$$$$$$$$$$$$$$$$$$$$$$$$$$$$$$$$$$
' Define Subroutines

Sub Heat_Eval
' This function evaluates if heat should occur to the plots.  It recovers data from the permanent memory
and uses this to determine if there is sufficient battery power and if the current temperature conditions
warrent powering the grids.  Note that timing is important here: this function is called on the fall of
EVAL_INTERVAL and that the call to Heat_Eval data table must happen before this call in order to provide
data to this function.  Summary table is called after and stores the resulting decision of this function
call.
      Const UNHEATED_OFFSET = 2            ' The first location of the unheated plots
      Const HEATED_OFFSET = 8              ' The first location of the heated plots
      Const ENERGY_OFFSET = 14             ' The first location of the energy output
      Const ENERGY_OFFSET_PLOTS = 17       ' The first location of the energy data for the plots we use

      Dim Heat_Data(25)                    ' Used to recover the necessary data from the Heat_Eval table
      Alias Heat_Data(1) = Batt_Voltage    ' The battery voltage
      Dim Heat_Avg                         ' Overall temperature and heat output averages
      Dim Warm_Temps(6)                    ' Warmed plot temperatures that meet shunt criteria
      Dim T_delta                          ' The difference between the heated and unheated plots (deg C)
      GetRecord (Heat_Data(),Heat_Eval,1) ' Recover the relevent data

      ' Process control temperature data: compute quality controlled averages
      AvgSpa (Heat_Avg,12,Heat_Data(ENERGY_OFFSET))    ' Heat output average of all plots
      For I = 0 To 5
            If (Heat_Data(I+ENERGY_OFFSET_PLOTS) >= Heat_Avg*VALID_HEAT ) Then
                  ' If the plot is being heated correctly then include it in the average...
                  Warm_Temps(I+1) = Heat_Data(HEATED_OFFSET+I)
            Else
                  ' ...Otherwise give NAN so will not be included in average
```

```
                Warm_Temps(I+1) = NAN
            EndIf
        Next I
        AvgSpa (T_cont_avg,6,Heat_Data(UNHEATED_OFFSET))' Compute the average.  AvgSpa ignores NAN values
        AvgSpa (T_warm_avg,6,Warm_Temps)
        If (T_warm_avg = NAN) Then
            AvgSpa (T_warm_avg,6,Heat_Data(HEATED_OFFSET))
        EndIf
        T_delta = T_warm_avg - T_cont_avg    ' Compute difference between averages

        ' Figure out if we should be heating; note this is only called if we are in the correct date range.
        Heat_Flag = FALSE                    ' Assume we aren't heating unless we get through positively
        If Batt_Voltage > BATT_V_MIN Then    ' If the battery voltage is good...
            If T_delta < T_DELTA_MAX Then ' ...and if the soil temperature needs it...
                Heat_Flag = TRUE             ' ...then heat.
            EndIf
        Else                                 ' If the voltage is too low, increment the low voltage counter
            Volt2low = Volt2low+1
        EndIf

        If T_delta < T_DELTA_MIN Then        ' Log temperature extremes
            Temp2low = Temp2low+1
        EndIf
        If T_delta >= T_DELTA_MAX Then
            Temp2high = Temp2high+1
        EndIf
EndSub


'$$$$$$$$$$$$$$$$$$$$$$$$$$$$$$$$$$$$$$$$$$$$$$$$$$$$$$$$$$$$$$$$$$$$$$
' Main Program
BeginProg
        ' Declare local variables
        Dim rTime(9)        ' Real time array (used to get the current day)
        Alias rTime(9) = Current_Day         ' Alias the current day
        If Duty_Cycle < DUTY_CYCLE_MIN Then Duty_Cycle = DUTY_CYCLE_DEFAULT ' In case it has been preserved

        Scan (EXEC_INTERVAL,Sec,0,0)
            PanelTemp (T_panel,250)          ' Measure panel temperature
```

```
Battery (V_logger)            ' Measure voltage output of DC/DC converter powering data logger
VoltDiff (V_battery,1,mV250,3,True ,0,250,VOLT_DIVIDER,0)    ' Measure main battery voltage

' Gather field data: thermocouples, shunts, solar charge current (can't autorange with PWM)
Dim T_TCref                   ' Reference temp on AM25T
AM25T (T_controls(),12,mV2_5C,1,1,TypeT,T_TCref,2,1,Vx1,True,1000,250,1.0,0)
AM25T (A_shunt(),12,mV250,13,1,-1,T_TCref,2,1,Vx1, True,0,_50Hz,0.001/R_SHUNT,0)
VoltDiff (A_solar,1,mV250,2,True, 0,_50Hz,0.001/R_SHUNT_SOLAR,0)

' Process shunt data; zero data if not heating to avoid noise (it confuses Heat_Eval)
For I = 1 To 12               ' Calculate the energy output to each plot and the solar array
      If (Heat_Flag) Then kJ_shunt(I) = A_shunt(I) * V_battery * EXEC_INTERVAL / 1000
      Else kJ_shunt(I) = kJ_shunt(I)*0     ' Multiplying by 0 preserves NAN values
Next I
kJ_solar = A_solar * V_battery * EXEC_INTERVAL / 1000 ' New charge/discharge

' If we are within the running dates run the optimization and heating code, otherwise ignore
it.  This means the optimization code will not mess with the Duty_Cycle during the winter, and
we can save memory space by not writing to the Heat_Eval and Daily_Report tables
RealTime (rTime)
If (Current_Day >= START_DAY) AND (Current_Day < END_DAY) Then
      CallTable Daily_Report  ' Write the daily summary data
      If TimeIntoInterval(OPTIMIZATION_TIME,24,Hr) Then
            Temp2low = 0      ' Reset counters
            Temp2high = 0
            Volt2low = 0
      EndIf

      ' If this is last EVAL_AVERAGING of the cycle enable averaging for evaluation data.  This
      eliminates transient data from past heatings being used in the evaluation routine.
      If TimeIntoInterval(EVAL_INTERVAL-EVAL_AVERAGING,EVAL_INTERVAL,Sec) Then
            Process_Disable = FALSE
      EndIf

      ' Determine if heating should occur. This needs to happen in the main loop because it
      needs to execute each EXEC_INTERVAL whereas Heat_Eval is only called each EVAL_INTERVAL.
      CallTable Heat_Eval           ' Write the data before we need to access it
      If TimeIntoInterval(0,EVAL_INTERVAL,Sec) Then
            Call Heat_Eval          ' Figure out if we should be heating the next EVAL_INTERVAL
```

```
                Process_Disable = TRUE  ' Ignoring data until final EVAL_AVERAGING of EVAL_INTERVAL
                Duty_Counter = 0        ' Mark the beginning of a new heating cycle
            EndIf
        Else        ' Outside date range so reset all counters
            Heat_Flag = FALSE               ' Be on the safe side here and reset these anyway
            Duty_Cycle = DUTY_CYCLE_DEFAULT ' Start the new year with a fresh duty cycle
            Temp2low = 0
            Temp2high = 0
            Volt2low = 0
            Duty_Counter = 0
        EndIf

        If Duty_Counter >= Ceiling(Duty_Cycle * EVAL_INTERVAL) Then
            Heat_Flag = FALSE               ' End of duty cycle so shut things down
        EndIf

        If Heat_Flag = FALSE Then
            PortSet (SWITCHER_PORT,0)     ' If Heat_Flag is low for whatever reason, turn off system
        Else
            PortSet (SWITCHER_PORT,1)     ' ...otherwise turn it on
        EndIf

        ' Final house keeping items
        CallTable Summary ' Write summary data. By doing it after the Heat_Eval block we capture
        whether the program intends to heat the next cycle.
        CallTable Raw     ' Note!: This table is for debugging purposes only and THIS LINE plus the
        TABLE DEFINITION should be commented out to give enough memory to get through the winter.

        Duty_Counter = Duty_Counter + EXEC_INTERVAL     ' Location of this increment is important --
        consider carefully before moving it.
    NextScan
EndProg
```
